# Well-being, problematic alcohol consumption and acute subjective drug effects in past-year ayahuasca users: a large, international, self-selecting online survey

**DOI:** 10.1038/s41598-017-14700-6

**Published:** 2017-11-09

**Authors:** Will Lawn, Jaime E. Hallak, Jose A. Crippa, Rafael Dos Santos, Lilla Porffy, Monica J. Barratt, Jason A. Ferris, Adam R. Winstock, Celia J. A. Morgan

**Affiliations:** 1 Clinical Psychopharmacology Unit, University College London, London, UK; 2 Psychopharmacology and Addiction Research Centre, University of Exeter, Exeter, UK; 3 Department of Psychiatry, University of Sao Paolo, Ribero, Preto, Brazil; 4 Institute for Social Science Research, University of Queensland, St Lucia, Australia; 5 Drug Policy Modelling Program, National Drug and Alcohol Research Centre, UNSW, Sydney, NSW Australia; 6 National Drug Research Institute, Faculty of Health Sciences, Curtin University, Perth, WA Australia; 7 Behaviours and Health Risks Program, Burnet Institute, Melbourne, VIC Australia; 8 Global Drug Survey Ltd, London, UK

## Abstract

Ayahuasca is a natural psychedelic brew, which contains dimethyltryptamine (DMT). Its potential as a psychiatric medicine has recently been demonstrated and its non-medical use around the world appears to be growing. We aimed to investigate well-being and problematic alcohol use in ayahuasca users, and ayahuasca’s subjective effects. An online, self-selecting, global survey examining patterns of drug use was conducted in 2015 and 2016 (n = 96,901). Questions were asked about: use of ayahuasca, lysergic acid diethylamide (LSD) and magic mushrooms; demographics, current well-being and past-year problematic alcohol use of past-year ayahuasca users and comparison drug users; and subjective effects of ayahuasca and comparison drugs. Ayahuasca users (n = 527) reported greater well-being than both classic psychedelic users (n = 18,138) and non-psychedelic drug-using respondents (n = 78,236). Ayahuasca users reported less problematic drinking than classic psychedelic users, although both groups reported greater problematic drinking than the other respondents. Ayahuasca’s acute subjective effects usually lasted for six hours and were most strongly felt one hour after consumption. Within our online, self-selecting survey, ayahuasca users reported better well-being than comparison groups and less problematic drinking than classic psychedelic users. Future longitudinal studies of international samples and randomised controlled trials are needed to dissect the effects of ayahuasca on these outcomes.

## Introduction

Ayahuasca (‘the vine of the soul’) is a powerfully psychoactive brew which is thought to have been used for several hundred years by indigenous people in the Amazon^1^. It is usually prepared by boiling the leaves of the *Psychotria viridis* bush, which contains N,N-dimethyltryptamine (DMT), and combining with the stems of the *Banisteriopsis caap*i vine, which contains the β-carboline alkaloids harmine, harmaline, and tetrahydroharmine, to create a thick brown liquid^2^. DMT is a tryptamine hallucinogen which is thought to act primarily at 5-HT_2A_ receptors but also at 5-HT_2C_ and 5-HT_1A_^3^. DMT is orally inactive due to its breakdown by monoamine oxidase activity in the gastrointestinal tract. The β-carboline alkaloids in the *Banisteriopsis caapi* vine are monoamine oxidase inhibitors (MAO-I) and therefore render orally administered DMT psychoactive^4^.

Ayahuasca is used by both indigenous tribes and by members of modern syncretic religious groups in and around the Amazon, including the Santo Daime and Uniao do Vegetal^2^. However, use is not confined to the Amazon, and there has been suggestion of a recent global increase in use of the drug^5^, particularly amongst tourists travelling to South America. Use is also suggested to have increased in Europe and the US amongst Western ‘neoShamans’^6^. The psychedelic state induced by ayahuasca often makes users reflect on personal concerns and memories^7^ and produces intense emotions^8^. These effects are highly valued by ayahuasca users who characterize the drug experience as similar to a psychotherapeutic intervention^7^. Emerging scientific evidence complements the users’ view of ayahuasca as therapeutic: a small, uncontrolled pilot study found that symptoms of depression reduced following consumption of the brew^9^. There is further suggestion of its use in the treatment of addiction^10–12^.

If the drug is to represent an important treatment, however, it is critical that its short and long-term effects are investigated and safety well established. Several observational studies have examined the long-term effects of regular ayahuasca use in the religious context. In this work, long-term ayahuasca use has not been found to impact on cognitive ability, produce addiction or worsen mental health problems^10,13–16^. In fact, some of these observational studies suggest that ayahuasca use is associated with less problematic alcohol and drug use, and better mental health and cognitive functioning^10,12,15^.

However, given the apparently burgeoning use of the drug worldwide and its potential therapeutic value, it is important to further investigate its positive and negative effects across a wider sample of individuals and a broader range of users across the globe. Although observational studies assessing the long-term effects of ayahuasca on mental health have being conducted in Brazil, United States, and Spain, the vast majority of this research has been limited to religious contexts. In particular, this study using a large, self-selecting, international sample, sought to examine the demographics of past-year ayahuasca users and subjective effects of ayahuasca in people who had recently used the drug compared to other hallucinogens as a reference (the classic psychedelics, LSD and psilocybin). In addition, based on previous studies suggesting positive effects of ayahuasca we aimed to compare current well-being, past year problematic drinking and lifetime diagnoses of mental illness of past-year ayahuasca users and in comparison to past-year users of other hallucinogenic drugs. We then also conducted an exploratory analysis to assess whether there were different associations between ayahuasca use and these outcomes in countries with and without a history of ayahuasca use. Based on previous observational research, we hypothesised that past-year ayahuasca users would have better well-being and less problematic drinking than comparison drug-using groups.

## Methods

### Design and participants

An international online cross-sectional drugs survey, the Global Drug Survey 2016 (GDS), was conducted between November 2015 and February 2016. GDS recruits participants by working in partnership with global media partners (e.g. advertising via Mixmag, The Guardian and Fairfax Media) and onward promotion through social media. This method has previously been employed to explore the use of uncommon and new drugs^17–19^. All participants were aged 16 years of age or over, none disclosed identifying information online, and all provided informed consent. Before taking the survey, potential respondents were made aware that the information they provide is anonymous and confidential, that you can withdraw at any time, that the GDS is the world’s largest annual drug survey, and that there is no financial incentive but that you are contributing to a better understanding of drug use around the world. Ethical approval was received from the Psychiatry, Nursing and Midwifery Research Ethics Subcommittee at King’s College London. The survey was conducted in line with the research ethics committee requirements.

### Assessments

The survey collected demographic data and detailed information on drug use. Respondents were asked about: their country of residence, gender (male, female, transgender), age, self-reported well-being, presence of a lifetime diagnosis of a mental illness and current problematic alcohol consumption. Self-reported well-being was measured using the Personal Wellbeing Index^20^, which has a maximum score of 80. Problematic alcohol use within the last year was measured using the Alcohol Use Disorder Identification Test (AUDIT)^21^, which has a maximum score of 40 and a hazardous drinking cutoff of >8. For this study, participants were asked about their ayahuasca use, and for comparison, their LSD and magic mushroom use (the two most commonly taken classic hallucinogens).

In order to compare demographic variables, current well-being, past year problematic drinking and lifetime mental illness we formed three groups: Ayahuasca Users (those who had taken ayahuasca in the last year), Classic Psychedelic Users (those who had taken either LSD or magic mushrooms in the last year, but not ayahuasca in the last year), and Other Respondents (those who had not taken ayahuasca, LSD or magic mushrooms in the last year). We combined past-year magic mushroom and past-year LSD users because these drugs are the two most popular serotonergic psychedelic drugs, have similar actions in the brain and we believe users of both drugs form a coherent group (evidence for this is reported in the results).

In addition, we stratified participants into those from countries with historical ayahuasca use and those from countries without historical ayahuasca. Countries considered to have a history of ayahuasca use were Bolivia, Brazil, Colombia, Ecuador, Peru and Venezuela. All other countries were considered not to have a history of ayahuasca use. We investigated whether differences between user groups (Ayahuasca, Classic Psychedelic, Other Respondents) existed in both countries with historical ayahuasca use and countries without historical ayahuasca use, or if they only existed on one type of country.

Moreover, respondents were asked to name the last new drug that they tried and answer questions about their subjective experience. We focused on respondents whose last new drug tried was ayahuasca, LSD or magic mushrooms. Specifically, they were asked to rate from 0 to 10: ‘the pleasurable high’, ‘the strength of the effect’, ‘the negative effects while high’, ‘the comedown after use’, ‘the urge to use more of the drugs when using’, ‘the value-for-money’ and ‘the risk of harm following a session of use’. Therefore, we could compare the subjective effects of ayahuasca with LSD and magic mushrooms, in new users. We only analysed data from people who reported taking ayahuasca and magic mushrooms orally or people who reported taking LSD either orally or sublingually. Furthermore, for ayahuasca, we report how the drug was sourced, how long the effects lasted, how long it took for ‘peak effects’ to occur after a single dose, and what the ‘predominant effect’ of the drug was.

### Statistical analyses

Descriptive statistics are provided for the demographic variables. Where data are missing, we report valid percentages, rather than absolute percentages.

We used one-way between-group ANOVAs and chi-square tests to compare the Ayahuasca User, Classic Psychedelic User and Other Respondent groups on age, gender, country of residence, past year problematic alcohol use, current well-being and lifetime mental illness diagnosis. In addition, we employed chi-square tests and two-way ANOVAs to investigate whether coming from a country with or without historical ayahuasca use moderated the above differences.

We used one-way between-group ANOVAs to compare the ratings of drug effects of ayahuasca, LSD and magic mushrooms in those whose last new drug tried was one of those.

If the groups had unequal variances then Welch’s tests (for one-way ANOVA) and t-tests allowing for unequal variances were used; this is reflected in the degrees of freedom reported where the second degree of freedom is not an integer. This was the case for all drug effect ANOVAs and many of the Ayahuasca/Psychedelic/Other Respondent group ANOVAs. Post-hoc t-tests and post-hoc z-tests (for proportions) were Bonferoni corrected. Data relevant to the results are available on request.

Data is available on request to other researchers who are interested in this manuscript.

## Results

During cleaning of the data, 3,817 records were excluded due to data capture glitches, duplicate entries, reporting no psychoactive drug use at all (including alcohol), reporting the use of a fake drug, and being 100 years of age or over.

### Whole sample demographics

A total of 100,711 responses were recorded. After exclusion of invalid responses, there were 96,901 responses remaining. The number of respondents from each country, that has at least 1,000 respondents, is shown in Table 1.

**Table 1:** The number of respondents in the sample from different countries. Only countries with n > 1,000 are shown.

Country	Number of respondents	% of sample
Germany	29,866	30.8
Switzerland	8,174	8.4
New Zealand	7,633	7.9
U.K.	6,015	6.2
U.S.A.	5,367	6.2
Netherlands	5,058	5.2
Australia	4,931	5.1
France	3,858	4.0
Italy	3,189	3.3
Hungary	3,071	3.2
Spain	2,520	2.6
Colombia	2,095	2.2
Austria	2,055	2.1
Norway	1,461	1.5
Canada	1,297	1.3
Mexico	1,203	1.2
Belgium	1,027	1.1

The majority of respondents were male (n = 62,584, 65.6%), 32,547 respondents were female (34.1%) and 449 were transgender (0.5%). The mean age of respondents was 28.71 (SD = 11.18).

A relatively small number of respondents reported lifetime ayahuasca use (n = 1,407, 1.5%) compared to over a quarter of the sample reporting lifetime magic mushroom or LSD use (n = 27,146, 28.0%; n = 25,953, 26.8%, respectively). Within the last year, 527 people (0.5%) reported ayahuasca use.

### Ayahuasca Users, Classic Psychedelic Users and Other Respondents

We split the respondents into: Ayahuasca Users (those who had used ayahuasca in the last year, n = 527), Classic Psychedelic Users (those who had used magic mushrooms and LSD, but not ayahuasca, in the last year, n = 18,138) and Other Respondents (other respondents who had not used any of these drugs in the last year, n = 78,236).

#### Drug use

*Use of commonly used recreational drugs in all three groups is reported in* Table 2*. There was a general pattern for more of the Classic Psychedelic Users and Ayahuasca Users to have used recreational drugs in their lifetime and in the last month than the Other Respondents*.

**Table 2:** The percentages and numbers of lifetime and last month drug use within Other Respondents (total n = 78,236), Classic Psychedelic Users (total n = 18,138) and Ayahuasca Users (total n = 527).

Drug	% Ever used (n)			% Used in last month (n)		
	Other Respondents	Classic Psychedelic	Ayahuasca	Other Respondents	Classic Psychedelic	Ayahuasca
Alcohol	97.9% (76,611)	98.5% (17,874)	97.5% (514)	86.5% (67,663)	90.4% (16,396)	87.1% (459)
Cannabis	76.7% (59,981)	97.6% (17,696)	94.9% (500)	41.3% (32,286)	83.3% (15,107)	78.7% (415)
Tobacco	75.1% (58,793)	84.4% (15,305)	79.5% (419)	48.5% (37,983)	67.1% (12,165)	59.8% (315)
Ecstasy/MDMA	34.3% (26,822)	80.9% (14,682)	71.5% (377)	10.0% (7,862)	38.4% (6,967)	24.7% (130)
Cocaine	27.5% (21,531)	58.5% (10,607)	61.5% (324)	7.3% (5,732)	22.7% (4,120)	17.6% (93)
Amphetamine	20.4% (15,975)	46.1% (8,358)	37.4% (197)	4.7% (3,662)	17.8% (3,233)	8.9% (47)
Magic Mushrooms	16.9% (13,204)	74.9% (13,539)	66% (348)	0% (0)	13.4% (2,434)	13.1% (69)
LSD	14.8% (11,584)	76.9% (13,953)	78.7% (415)	0% (0)	21.4% (3,879)	21.6% (114)
Methamphetamine	4.1% (3,179)	9.5% (1,725)	11.2% (59)	0.5% (410)	1.8% (321)	0.9% (5)
Heroin	3.0% (2,308)	5.3% (953)	7.2% (38)	0.4% (310)	1.1% (197)	0.8% (4)
Ayahuasca	0.5% (419)	2.5% (461)	100% (527)	0% (0)	0% (0)	18.2% (96)

#### Demographic variables

There was a main effect of user group on age (F_2, 1377.17_ = 122.24, p < 0.001). Ayahuasca Users (mean = 29.09, SD = 10.03, n = 515) were older than the Classic Psychedelic Users (mean = 24.17, SD = 6.79, n = 17,937) (t_527.56_ = 11.06, p < 0.001). The Ayahuasca Users had a similar age to the Other Respondents (mean = 29.76, SD = 11.73, n = 77,353) (t_523.39_ = 1.52, p = 0.26).

Fewer females than males reported ayahuasca use. Genders were not split similarly across the groups (χ^2^(4) = 928, p < 0.001). The proportion of females in the Ayahuasca User group (n = 146, 28.0%) was similar to the Classic Psychedelic User group (n = 4,380, 24.5%) but smaller than in Other Respondents (n = 28,021, 36.3%) (z = 3.92, p < 0.001).

**Figure 1:**
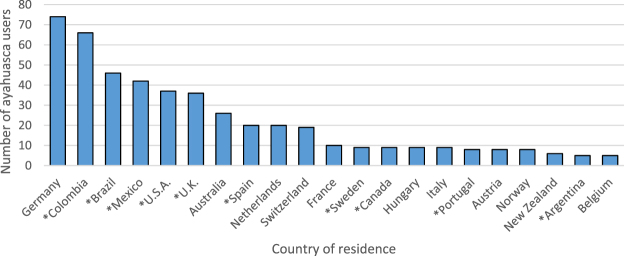
The number of ayahuasca users (people who have taken ayahuasca in the last year) in different countries. *Designates countries where there were more ayahuasca users than expected by chance, relative to classic psychedelic users and other respondents. Only countries with n ≥ 5 ayahuasca users are shown.

In terms of geographical locations, some countries had more ayahuasca users (relative to the other two groups) than expected by chance: Argentina, Brazil, Canada, Colombia, Mexico, Portugal, Spain, Sweden, the U.K. and the U.S.A. The number of ayahuasca users in each country is shown in Fig. 1.

#### Psychological Well-Being

**Figure 2:**
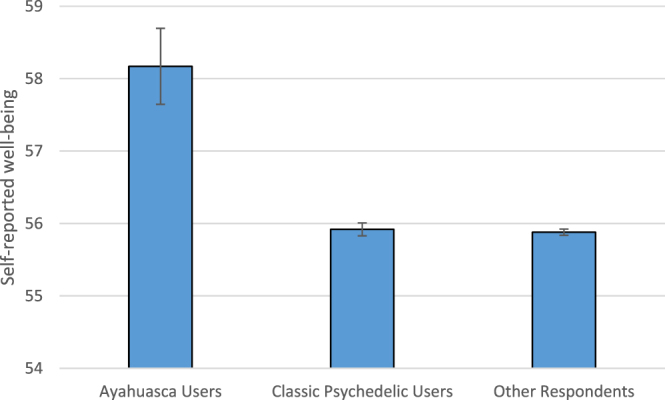
*Bar chart of self-reported well-being on the* Personal Wellbeing Index^20^, which has a maximum score of 80. Bars show mean scores. Error bars show standard error scores.

There was a main (Fig. 2) effect of user group on reported well-being (F_2, 1372.22_ = 10.70, p < 0.001). The Ayahuasca Users (mean = 58.17, SD = 11.98, n = 521) reported greater well-being than the Classic Psychedelic Users (mean = 55.92, SD = 11.91, n = 17,893) (t_550.364_ = 4.217, p < 0.001) and the Other Respondents (mean = 55.88, SD = 12.07, n = 77,473) (t_527.124_ = 4.329, p < 0.001).

#### Past Year Problematic Alcohol Use

There was a main effect of user group on total AUDIT score (F_2, 1777.96_ = 692.36, p < 0.001). The Ayahuasca User group (mean = 9.41, SD = 5.91, n = 458) had a lower total AUDIT score than the Classic Psychedelic User group (mean = 10.33, SD = 5.94, n = 16,615) (t_17071_ = 3.216, p = 0.002). However, the Ayahuasca User group had a higher total AUDIT score than the Other Respondents (mean = 8.45, SD = 5.50, n = 68,775) (t_69231_ = 3.73, p < 0.001). Importantly, all three groups would meet the hazardous drinking criterion of >8.

#### Lifetime Mental Illness Diagnoses

The proportion of people who reported having a diagnosed mental illness in their lifetime was not similar across the groups (χ^2^(2) = 133.36, p < 0.001). There was a similar proportion of people who reported having ever been diagnosed with a mental illness in the Ayahuasca User group (n = 129, 24.9%) and the Classic Psychedelic User group (n = 4,014, 22.4%), but a larger proportion in the Ayahuasca User group compared with the Other Respondents (n = 14,549, 18.8%) (z = 5.48, p < 0.001).

#### Differences within Classic Psychedelic Users

We grouped magic mushroom and LSD users together as a comparison group. Evidence that respondents who used LSD in the last year but did not use magic mushrooms in the last year are similar to those who used magic mushrooms in the last year but did not use LSD in the last year is as follows: these groups did not differ on age (p = 0.270), wellbeing (p = 0.699), problematic drinking (p = 0.319) and gender (p = 0.962). However, the only last-year LSD users were slightly more likely to have a lifetime mental illness diagnosis than the only last-year magic mushroom users (χ^2^(1) = 5.113, p = 0.024).

### Exploratory analysis of countries with and without a history of ayahuasca use

Given our findings above, we explored whether being from a country with a history of ayahuasca use moderated the relationship between ayahuasca use and various outcomes.

#### Psychological Well-being

There was an interaction between User Group and Country Type on well-being (F_2, 690.98_ = 4.77, p = 0.009). Post-hoc t-tests showed that, within the countries with a history of ayahuasca use, there were no differences in psychological wellbeing between the user groups. However, within the countries without a history of ayahuasca use, Ayahuasca Users had a higher well-being (mean = 58.66, SD = 11.95, n = 406) than Classic Psychedelic Users (mean = 55.91, SD = 11.87, n = 16,723) (t_2.74_, p < 0.001) and Other Respondents (mean = 55.86, SD = 12.06, n = 77,460) (t_2.80_, p < 0.001).

#### Lifetime Mental Illness Diagnoses

We found that amongst countries with a history of ayahuasca use there were no differences in incidence of lifetime mental illness diagnoses between Ayahuasca Users, Classic Psychedelic Users and Other Respondents. However in countries without a history of ayahuasca use there was a significant difference between incidence of lifetime mental illness diagnoses (χ^2^ (1) = 168.05, p < 0.001). Ayahuasca Users had a significantly greater history of mental illness diagnoses (n = 114, 28.3%) than Classic Psychedelic Users (n = 3,849, 23%) who in turn had a significantly greater lifetime incidence than Other Respondents (n = 14,262, 18.9%).

#### Past Year Problematic Use

There was no interaction between User Group and Country Type for AUDIT scores.

### For those who said ayahuasca was their most recently tried new drug

192 people said ayahuasca was the drug they had most recently tried for the first time. Of these, 191 reported their route of administration: 166 people (86.9%) swallowed it, 20 people (10.5%) said ‘other’, which equated to ‘swallowed’ (e.g. ‘drank it’), and 5 people (2.6%) said they smoked it. As stated above, only respondents who reported taking ayahuasca orally (i.e. ‘swallow’ or ‘other’) were included in these analyses. As smoking ayahuasca is not a viable route of administration, these respondents were excluded in these analyses.

50 people (26.9%) had sourced the ayahuasca from a friend, 13 (7.0%) from a website, 5 (2.7%) from a family member, 3 (1.6%) from a dealer, 1 (0.5%) from a headshop and 114 (61.3%) from ‘other’. Of those who said ‘other’, there were a variety of responses, but apart from 3 (‘Holland’, ‘Organisation’, ‘Self-Brewed’), these all referred, in essence, to a ‘shaman’ or a ‘healer’. Examples include: ‘Healing centre’, ‘At Retreat’, ‘Ceremony’, ‘Church’, ‘Santo Daime’, ‘Teacher’, ‘Trained Facilitator’.

51 people (27.4%) said they had already taken it again since the first time; 86 people (46.2%) said they planned to take it again but had not yet; and 15 people (8.1%) said they did not plan to take it again.

### Duration of effect

**Figure 3:**
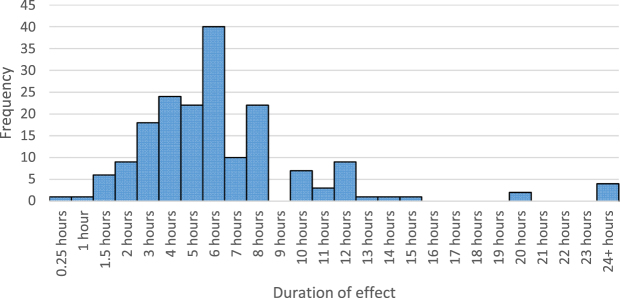
The number of people who reported ayahuasca’s duration of effect, from 0.25 hours to 24+ hours (n = 181). Only people whose last new drug tried was ayahuasca answered this question.

The median and modal reported duration of effect was 6 hours (n = 40) (see Fig. 3), although there was some positive skew with 4 people reporting a duration of effect of 24+ hours.

### Time to peak effect

**Figure 4:**
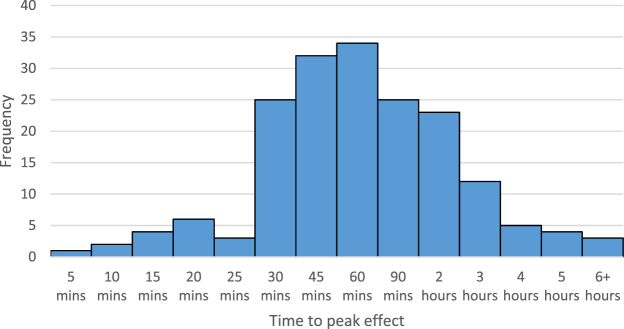
The number of people who reported ayahuasca’s time to peak effect, from 10 mins to 6+ hours (n = 179). Only people whose last new drug tried was ayahuasca answered this question.

The median and modal estimated time to peak effect was 1 hour (n = 34) (see Fig. 4).

### Subjective effects

Most people (n = 131, 70.8%) described ayahuasca as ‘mostly psychedelic’, three people (1.6%) described it as ‘mostly cannabis like’, two people (1.1%) described it as ‘mostly empathogen like’, one person described it as ‘mostly stimulant like’ and 48 people (25.9%) stated ‘other’. Of those that said ‘other’, most described it as (or something similar to): ‘spiritual’, ‘medicine’, ‘healing’, ‘hallucinogenic’, ‘indescribable’. Two people reported ‘feeling nothing’. Other responses (of n = 1) included: ‘crazy’, ‘nausea’, ‘introspective’.

Ratings of the effects of ayahuasca/LSD/magic mushrooms were reported by people whose last new drug they had tried was ayahuasca/LSD/magic mushrooms, respectively (see Table 3). The strength of effect of ayahuasca was rated as greater than LSD (t_189.22_ = 189.22, p = 0.014) and magic mushrooms (t_196.51_ = 7.94, p < 0.001). The pleasurable effects of ayahuasca were rated as smaller than LSD (t_184.337_ = 2.66, p = 0.018). The negative effects while high on ayahausca were rated as greater than LSD (t_184.07_ = 3.382, p = 0.002) and Magic Mushrooms (t_186.87_ = 4.82, p < 0.001). The comedown after ayahuasca was rated as smaller than LSD (t_191.04_ = 6.60, p < 0.001) and Magic Mushrooms (t_191.68_ = 2.39, p = 0.034). The urge to use more of ayahuasca was rated as smaller than LSD (t_199.52_ = 4.46, p < 0.001) and Magic Mushrooms (t_206.58_ = 4.56, p < 0.001). The risk of harm following a session of ayahuasca was rated as lower than LSD (t_204.05_ = 8.49, p < 0.001) and magic mushrooms (t_204.31_ = 4.98, p < 0.001). Finally, there were no significant differences between ratings of value for money between Ayahuasca and the other drugs.

**Table 3:** Drug effect profiles of ayahuasca, LSD and magic mushrooms, among subsamples who reported recently trying these drugs for the first time. Mean (SD).

	Ayahuasca	LSD	Magic Mushrooms	F _(df1, df2)_
The pleasurable high	7.15 (2.82), n = 178	7.72 (2.13), n = 4944	7.14 (2.27), n = 3844	76.22 _(2, 472.11)_***
Strength of effect	8.26 (2.19), n = 179	7.80 (2.03), n = 4935	6.92 (2.29), n = 3828	187.88 _(2, 481.22)_***
Negative effects when high	3.88 (3.15), n = 176	3.07 (2.64), n = 4842	2.72 (2.66), n = 3741	26.480 _(2, 469.47)_***
Comedown after use	2.15 (2.65), n = 177	3.49 (2.83), n = 4837	2.63 (2.54), n = 3744	117.86 _(2, 478.70)_***
Urge to use more when intoxicated	0.8 (1.71), n = 178	1.39 (2.23), n = 4878	1.41 (2.24), n = 3790	10.60 _(2, 493.32)_***
Value-for-money	7.57 (2.90), n = 176	7.79 (2.46), n = 4819	7.26 (2.69), n = 3718	44.19 _(2, 470.18)_***
Risk of harm following a session of use	1.44 (1.96), n = 179	2.72 (2.72), n = 4888	2.19 (2.41), n = 3800	69.63 _(2, 497.14)_***

Ns vary slightly for each drug effect rating due to missing data. ***p < 0.001. Welch’s ANOVAs were used due to a violation of the homogeneity of variance assumption.

## Discussion

This study aimed to investigate the characteristics of past-year ayahuasca users (who will be referred to simply as ‘ayahuasca users’), their current well-being, their past year problematic alcohol use and their lifetime mental illness diagnoses relative to comparison drug user groups, and the subjective effects of ayahuasca in new users. Given prior observational research with individuals taking the drug in a religious context, we hypothesised that ayahuasca users would have better current well-being and less past year problematic drinking.

In the current study, ayahuasca users reported greater well-being than past-year LSD and magic mushroom users (who will be referred to simply as ‘classic psychedelic users’) and the non-psychedelic using other respondents. Ayahuasca users reported less problematic drinking than classic psychedelic drug users. However, both groups would be considered hazardous drinkers according to the AUDIT cut-off and both groups reported higher greater problematic drinking than the other respondents in this survey. Ayahuasca and classic psychedelic users were also more likely to report lifetime diagnoses of mental health problems than other survey respondents.

We also examined the subjective effects of ayahuasca in people whose most recently taken new drug was ayahuasca. The modal and median duration of effect of ayahuasca was 6 hours and the modal and median time to peak effect was 1 hour. Generally, compared to LSD and magic mushrooms, ayahuasca was rated as: stronger; less pleasurable; having more negative effects while high; having weaker ‘comedown’ effects after use; creating less of an urge to use more; and having less risk of harm following a session of use. Ayahuasca users were more likely to live in countries in South America, particularly Colombia (n = 66) and Brazil (n = 46). The vast majority of new ayahuasca users ingested the drug via swallowing or drinking, and most acquired the drug from a shaman or a ‘healer’. Across the whole survey, ayahuasca use was also substantially less common than use of LSD or magic mushrooms. However, it is important to note that the survey was self-selecting and so these values do not reflect true estimates of the proportions of people worldwide who use these drugs.

Self-rated psychological well-being was better in ayahuasca users than either classic psychedelic users or other respondents in the survey. This finding fits with previous studies that have found beneficial effects of ayahuasca use on subjective well-being^13,22^. It is of particular interest that ayahuasca users had better well-being than classic psychedelic users. DMT (the main active compound in ayahuasca), LSD and psilocybin (the active compound in magic mushrooms) all cause their psychedelic effects primarily via agonism of the 5-HT_2A_ receptor^23,24^. Therefore, some other pharmacological difference, perhaps due to the non-DMT compounds in ayahuasca; a difference in the way the drugs are taken; or a pre-existing difference in the kinds of people who use ayahuasca compared with LSD and magic mushrooms, may explain this finding. The ceremonial nature of ayahuasca consumption, and the supporting community, may contribute to its positive effects on well-being. Interestingly, these data do not replicate findings that the use of classic psychedelic drugs is associated with greater well-being in the long-term^25,26^, as this effect was not observed in the classic psychedelic users compared to other respondents. This may well be a result of confounding factors, such as additional drug use and pre-existing differences between classic psychedelic drug users and the other respondents in this survey. Furthermore, in Griffith *et al*.’s studies, participants are usually hallucinogen-naïve, often report regular participation in religious or spiritual activities, are screened for mental health problems, are prepared before drug administration, are followed during and after drug effects, and the drug is administered in a controlled, laboratory environment under comfortable, supportive conditions. These characteristics may reduce adverse reactions, and are usually not present in uncontrolled settings.

This study also found a higher incidence of lifetime mental health problems in both ayahuasca users and classic psychedelic users, compared to the non-psychedelic other respondents. A previous study of ayahuasca users from Brazil, who were using the drug in a religious context, found lower self-rated psychopathology maintained over one year compared to non-drug using controls^13^. A limitation of our study was that *current* mental health diagnoses or psychopathology was not measured, so we cannot distinguish between rates of current or resolved mental health conditions. It may be that people who use more drugs, more frequently (i.e. the ayahuasca and classic psychedelic user groups engaging in poly-drug use) are likely to have more lifetime mental illness diagnoses than those who use less drugs, less frequently (i.e. the other respondents), unrelated to ayahuasca consumption. This could be a result of mental illness preceding poly-drug use, or a result of poly-drug use preceding mental illness diagnosis. An alternative explanation for this relationship may be that people who have or have had mental health problems may be trying or have tried ayahuasca and/or other psychedelic drugs as an unconventional treatment.

When we stratified by country of origin (with or without historical ayahuasca use), we found that lifetime mental health diagnoses were no different amongst users from countries with a history of ayahuasca use. Contrastingly, in those countries without traditional or religious ayahuasca use, people who used ayahuasca had a higher incidence of lifetime mental health diagnoses than either classic psychedelic users or other drug users. We might speculate that different kinds of people may be attracted to ayahuasca for different reasons in countries with and without historical ayahuasca use. For instance, in countries where ayahuasca has been historically used and is more socially accepted today, the experience may be considered more normal and therefore appeal to the mainstream. In contrast, in countries in which it is illegal to possess, use must be clandestine and confined to certain sub-cultures. People who engage in such illicit behaviours are more likely to have lifetime mental health diagnoses, which may contribute to our finding. Previous work has examined the impact of cultural context on the response to drugs and expectation of their positive and negative effects^27^. The expectation of negative effects from a drug is likely greater in a country where narratives of psychedelic experiences as therapeutic are largely absent^28^; this could in turn drive more negative psychedelic experiences and possibly contribute to mental health problems.

Ayahuasca has recently emerged as a promising treatment for depression^29^, and longitudinal studies of users have found lower incidence of mental health issues associated with use of the drug^13^. In countries without a history of ayahuasca use, we found a higher incidence of lifetime mental illness diagnoses in the ayahuasca users compared with the classical psychedelic users and other respondents. However, we did not measure current mental health issues and so we cannot know whether ayahuasca use is associated with more or fewer current mental health problems. Future research should establish the direction of causality, i.e. are those with mental health problems drawn to seek out ayahuasca as a potential treatment? This explanation is congruent with the majority of the participants in this study reporting taking the substance in a healing context, from a shaman or other spiritual healer. If this were the case, it would not be incompatible with our finding of higher *current* ratings of well-being in the ayahuasca using group, relative to the other groups. However, future longitudinal studies are required to fully address this question.

Problematic drinking, as measured by the AUDIT, was less prevalent in ayahuasca users than the group using classic psychedelics. This is an interesting finding, as classic psychedelic users were similar to ayahuasca users in many other respects, and is concordant with previous observations of a reduction in substance use with regular ayahuasca use^30^. However, ayahuasca users did show greater problematic drinking than non-psychedelic using respondents in the sample. Moreover, although the groups were statistically different on this variable, the mean differences were relatively small. Whilst in this study it was necessary for us to compare our ayahuasca users to this broad and heterogenous sample of drug users, future work should aim to match more closely a comparison group. There were differences in drug use across these samples, such that the ayahuasca and classic psychedelic users consumed more substances in the previous year than the other respondents did. Therefore, given people who take ayahuasca are, on average, more likely to use many different drugs than the other respondents (as shown in Table 2), it is perhaps unsurprising that they have higher problematic drinking levels. Since ayahuasca appears to have anti-addictive potentials, which appear to be especially significant for alcohol^30^, this result could alternatively suggest that users could be seeking ayahuasca to treat their alcohol-related problems, but it is not possible to explore these interpretations due to our cross-sectional design. In order to examine the effects of ayahuasca use on alcohol use disorders more carefully, large longitudinal surveys and randomised controlled trials must be employed.

Ayahuasca produced a low urge to use the substance again, which is in agreement with previous findings^10^. In comparison to the classic psychedelics, themselves of a low-abuse potential^31^, ayahuasca engendered less desire to take more of the drug. These findings suggest that ayahuasca has a very low abuse potential, which speaks to its safety as an emerging treatment for depression, anxiety and drug addiction. Ayahuasca was rated as having stronger negative effects while high than LSD or magic mushrooms, and these differences were the largest observed. This may well be related to the well-known vomit-inducing effects it has. Interestingly, ayahuasca was rated as being stronger, but also less pleasurable. One might speculate that the ayahuasca experience is cultivated to be less about ‘pleasure’ and more about meaning, spirituality and learning, than LSD and magic mushrooms, which are frequently taken in a recreational way.

### Strengths and Limitations

One important strength of this study was the use of an international sample of drug users, making this the largest characterisation of ayahuasca use to date, and in the most demographically broad sample. This study, unlike previous work, was not confined to individuals who take the drug alongside membership of a syncretic church, and therefore did not confound the effects of the drug with the non-specific effects of being part of a specific religious organisation. Most importantly, our survey was self-selecting and so was not representative of the global population or the drug-using global population. Therefore, the conclusions we draw may not extend to all ayahuasca and classic psychedelic (LSD and magic mushroom) users. Moreover, we were reliant on self-report data and are unable to confirm the substance ingested was indeed ayahuasca, although the relatively large agreement in the profile of effects would suggest this was the case. It would have been useful to have data on number of lifetime ayahuasca sessions; we would then have been able to explore associations between extent of ayahuasca use and our outcomes. However, because the Global Drug Survey has a very large number of questions, questions about lifetime drug use were sacrificed in order to not overburden the respondents. Another limitation of the current study was that mental illness diagnoses were only measured over the lifetime, so it was not possible to discern current from resolved mental health problems. Future studies should therefore measure this variable.

## Conclusions

Ayahuasca users in this international mostly took the drug with a healer or a shaman. Ayahuasca was rated as less pleasant and with less of an urge to use more of it than classic psychedelics. We found evidence of greater well-being and less problematic alcohol use in ayahuasca users compared to classic psychedelic users. However, the ayahuasca users did have greater problematic drinking than the non-psychedelic using respondents. There was a higher incidence of lifetime mental illness diagnoses within the ayahuasca users, which subsequent analyses found to be confined to users from countries without a tradition of ayahuasca use. There is a clear need for more research into the relationships between ayahuasca use, mental health, well-being and problematic alcohol and substance use, within this heterogeneous group of users. Nevertheless, our findings of increased well-being and less problematic alcohol use amongst ayahuasca users, compared to their classic psychedelic using counterparts, lends limited support to the notion that ayahuasca may prove to be an important and powerful adjunct for the treatment of depression and alcohol use disorders. Well controlled studies of ayahuasca must be therefore be conducted in order to rigorously test its safety and efficacy within psychiatric disorders.
