# Author Correction: Well-being, problematic alcohol consumption and acute subjective drug effects in past-year ayahuasca users: a large, international, self-selecting online survey

**DOI:** 10.1038/s41598-018-21666-6

**Published:** 2018-03-01

**Authors:** Will Lawn, Jaime E. Hallak, Jose A. Crippa, Rafael Dos Santos, Lilla Porffy, Monica J. Barratt, Jason A. Ferris, Adam R. Winstock, Celia J. A. Morgan

**Affiliations:** 1 Clinical Psychopharmacology Unit, University College London, London, UK; 2 Psychopharmacology and Addiction Research Centre, University of Exeter, Exeter, UK; 3 Department of Psychiatry, University of Sao Paolo, Ribero, Preto Brazil; 4 Institute for Social Science Research, University of Queensland, St Lucia, Australia; 5 Drug Policy Modelling Program, National Drug and Alcohol Research Centre, UNSW, Sydney, NSW Australia; 6 National Drug Research Institute, Faculty of Health Sciences, Curtin University, Perth, WA Australia; 7 Behaviours and Health Risks Program, Burnet Institute, Melbourne, VIC Australia; 8 Global Drug Survey Ltd, London, UK

Correction to: *Scientific Reports* 10.1038/s41598-017-14700-6, published online 09 November 2017

The Acknowledgements section in this Article was omitted. The Acknowledgements section should read:

“This work was funded by a Medical Research Council UK Grant to CJAM (*MR*/*L023032*/*1*).”

